# Putative Role of an ABC Efflux System in *Aliarcobacter butzleri* Resistance and Virulence

**DOI:** 10.3390/antibiotics12020339

**Published:** 2023-02-06

**Authors:** Inês Martins, Cristiana Mateus, Fernanda Domingues, Mónica Oleastro, Susana Ferreira

**Affiliations:** 1 CICS-UBI—Health Sciences Research Centre, University of Beira Interior, 6201-506 Covilhã, Portugal; 2 National Reference Laboratory for Gastrointestinal Infections, Department of Infectious Diseases, National Institute of Health Dr. Ricardo Jorge, Av. Padre Cruz, 1649-016 Lisbon, Portugal

**Keywords:** *Aliarcobacter butzleri*, ABC efflux pumps, resistance, virulence, YbhFSR

## Abstract

*Aliarcobacter butzleri* is considered a ubiquitous microorganism and emergent pathogen, for which increasing rates of multidrug resistance have been described. In line with this, the present work aimed to evaluate for the first time the contribution of an ABC efflux system, the YbhFSR, in the resistance and virulence of this bacterium. Following the in silico characterization of the YbhFSR transporter, a mutant strain was constructed by inactivating the gene responsible for ATP-binding. After ensuring that the mutation did not have an impact on bacterial growth, the resistance profile of parental and mutant strains to different antimicrobial agents was evaluated. The results suggest that the efflux pump may influence the resistance to benzalkonium chloride, ethidium bromide, and cadmium, and several other compounds were identified as potential substrates. Regarding the evaluation of the accumulation of ethidium bromide, a slight increase was observed for the mutant strain, demonstrating a potential role of the YbhFSR efflux pump in the extrusion of toxic compounds from *A. butzleri*. Subsequently, the role of this efflux pump on the *A. butzleri* known virulence properties was evaluated, but no difference was seen among mutant and parental strains for the motility, biofilm formation ability, susceptibility to oxidative stress, or the ability to adhere and invade Caco-2 cells. However, in contrast to the parental strain, the mutant strain showed a resistance to human serum. Overall, the results support the role of efflux pumps in *A. butzleri* resistance to antimicrobials, highlighting the particular role of the YbhFSR system.

## 1. Introduction

*Aliarcobacter butzleri* is a Gram-negative bacterium, isolated from different environments and hosts, which, together with eight other distinct species, composes the genus *Aliarcobacter*, currently belonging to the order *Campylobacterales* and family *Arcobacteraceae* [1,2]. Classified as an emerging foodborne, waterborne and zoonotic enteropathogen [3,4], *A. butzleri* is one of the most relevant *Aliarcobacter* species for public health due to its association with animal and human disease [1]. In its relationship with human disease, it is typically associated with gastrointestinal disorders, such as enteritis or colitis, whose main symptoms are persistent watery diarrhea, abdominal cramps, nausea, and vomiting, while it occasionally may be associated with bacteremia or even with septicemia. Apart from gastrointestinal problems, in animals, mastitis and abortions cases resulting from infections by this microorganism are also reported [1,3]. In addition to its pathogenic potential, *A. butzleri* isolates have demonstrated a resistance to different antibiotic classes, including those most frequently used in the treatment of this infection, with multidrug resistance rates ranging from 20 to 93.8% being reported currently in the isolates from animals, humans, food products, and the environment [5,6,7,8,9,10,11,12].

However, and despite *A. butzleri* resistance being widely described and its virulence factors being increasingly studied, there is still a clear lack of information about the involved mechanisms. In addition to the identification of a target modification associated with fluoroquinolone resistance [13], the role of efflux pumps in the phenotype of antimicrobial resistance has recently been described [7,14,15]. Although the role of efflux pumps in antibiotic resistance is well established, the position that these structures may have in virulence, such as colonization and the survival of the bacteria in the host, is being increasingly recognized [15,16]. Efflux pumps can be grouped into six clinically relevant families: the major facilitator superfamily (MFS), small multidrug resistance (SMR) family, multidrug and toxic compound extrusion (MATE) family, proteobacterial antimicrobial compound efflux (PACE) family, resistance–nodulation–cell division (RND) family, and ATP-binding cassette (ABC) family [17].

The efflux pumps from the ABC family are able to recognize and transport a wide variety of substances, including sugars, amino acids, ions, drugs, polysaccharides, and proteins [18]. The particularity of the ABC-type transporters in relation to the other families of efflux pumps lies on their similar topology among different bacterial genus, being composed of two transmembrane domains (TMD), which usually form the passageway of the substrate, and two cytoplasmic nucleotide-binding domains (NBD), which are responsible for the energetic process of the pump. Thus, by the binding and hydrolysis of ATP and the consequent release of ADP, conformational changes are induced in the NBD, whose mechanical energy is transmitted to the TMD in order to extrude the substrate through the membrane [19,20]. In bacteria, these efflux pumps often have a TMD fused to an NBD and can form homodimers or heterodimers, depending on the bacterial species in question. Unlike NBD, TMD show some structural variability among ABC transporters, whether in their primary sequence, size, architecture, or number of transmembrane helices [19]. Although the number of transmembrane helices may vary between 8 and 20 for importers and 12 for exporters of the ABC-type, 6 transmembrane segments are typically described in exporters of this type [21,22,23]. In addition, NBD are highly conserved among ABC transporters and display Walker A and B motifs and a specific signature motif that defines the ABC superfamily [19].

ABC drug efflux pumps include single drug resistance efflux pumps and multidrug resistance efflux pumps [24]. The first characterized transporter in this superfamily was LmrA from *Lactococcus lactis*, which when cloned in *Escherichia coli,* was associated with a resistance to a wide range of compounds, such as ethidium bromide, rhodamine 6 G, and daunorubicin [25]. Since then, various ABC-type efflux pumps have been studied and described as conferring a resistance phenotype to several structurally unrelated compounds in different bacterial species [26,27,28,29].

The comparative genomic analysis performed by Isidro and collaborators (2020) in 49 *A. butzleri* genomes detected the presence of 19 putative efflux systems and the existence of 56 genes coding for efflux pumps [7]. Among them, three putative efflux systems belonging to the ABC family were identified, named as EP5, EP6, and EP10 [7], with the latter showing genetic homology with the previously described YbhGFSR transporter in *E. coli* [30]. Having observed that some of the genes are organized in operons with homology to ABC-type efflux transporters, an operon was selected for analysis in the present research. The operon in question, previously named EP10, is now designated *ybhGFSR* and includes the gene that codes for the membrane fusion protein YbhG and three genes coding for the YbhFSR transporter, the *ybhF* gene with 1701 bp, the *ybhS* gene with 1089 bp, and the *ybhR* gene with 1092 bp [7,30]. The active role of this transporter in tetracycline efflux and Na^+^(Li^+^)/H^+^ transport in *E. coli* was previously demonstrated [31].

Therefore, the main aim of this work was to contribute to a better understanding of the *A. butzleri* resistance and virulence mechanisms, focusing for the first time on the study of the role of an ABC-type transporter.

## 2. Results

### 2.1. Bioinformatic Analysis of the YbhFSR Efflux System

The SOSUIGramN software allowed for identifying the YbhF protein as cytoplasmic, and the YbhS and YbhR proteins as inner membrane proteins. The DeepTMHMM program demonstrated the absence of transmembrane domains encoded by the YbhF protein, contrasting with the presence of six α-helical transmembrane segments in the YbhS and YbhR proteins (Figure S1a), suggesting that the YbhFSR transporter forms the structural units ({TMD}_2_-{NBD}_2_), which is the characteristic structure of ABC-type efflux pumps.

The physicochemical properties of the 566 amino acids of the YbhF protein of *A. butzleri* were evaluated using the Protparam software, having a molecular weight of 63.58 kDa and a theoretical isoelectric point of 5.97. Considering the amino acid sequence, the Batch Web CD-Search tool software predicted YbhF as an export protein belonging to the family of ABC transporters.

Typically, the ATP-binding subunits contain conserved sequence motifs important for the transporter functions. In line with this, the presence of two ATP-binding domains in the YbhF from *E. coli* has been described [30]. By analyzing the conserved domains of this protein from *A. butzleri* using the online tool of the NCBI platform Conserved Domains, it was possible to identify the conserved domains of Walker A, Q-loop and Walker B, and the D-loop signature motifs and the Switch motif (Figure S1b).

The data provided by the software used in this in silico analysis allowed for the identification of the *A. butzleri* YbhFSR efflux pump as a potential ABC family transporter.

### 2.2. Influence of Efflux System Disruption in Bacterial Growth

**Figure 1:**
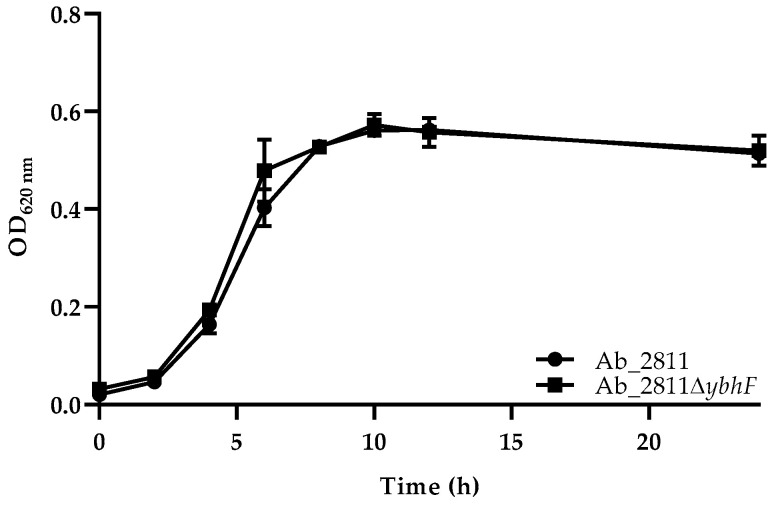
Growth curves of the parental strain *Aliarcobacter butzleri* Ab_2811 and derived mutant strain Ab_2811∆*ybhF*. Data correspond to mean ± standard deviation (SD) of two independent trials.

Once the disruption of the *ybhF* gene was confirmed through the incorporation of the constructed transformant DNA fragment in the genome of *A. butzleri* Ab_2811 by natural transformation (Figure S2), originating the Ab_2811Δ*ybhF* mutant strain, it was important to understand the effect of the efflux system’s impairment in bacterial growth. In this sense, the growth curves of the parental and the mutant strains under study were constructed (Figure 1). Both strains exhibited a similar growth curve, with no significant growth changes observed.

### 2.3. Impact of the Inactivation of YbhFSR Efflux Pump on Aliarcobacter butzleri Antimicrobial Resistance

The contribution of the YbhFSR efflux pump to *A. butzleri* resistance was firstly assessed by determining the minimum inhibitory concentration (MIC) of parental and mutant strains to several distinct antimicrobial agents, including antibiotics, disinfectants, germicide, efflux pump substrate, and metals (Table 1 and Table 2). These experiments allowed not only to assess the influence of the inactivation of the YbhFSR efflux pump on the resistance of this bacterium, but also to infer about the potential substrates of this pump. In general, the increased susceptibility of the mutant strain Ab_2811∆*ybhF*, compared to the parent strain *A. butzleri* Ab_2811, to several tested compounds was noticeable.

The differences observed between the parental and mutant strains suggest that among the 12 compounds tested, benzalkonium chloride and ethidium bromide are substrates of the YbhFSR efflux pump, for which the MIC values were notoriously reduced in the mutant strain when compared to the values obtained for the parental strain *A. butzleri* Ab_2811 (four-fold decrease in the MIC) (Table 1). Moreover, ampicillin, erythromycin, chloramphenicol, and acriflavine may also be potential substrates of the YbhFSR system, despite only a two-fold decrease in the respective MIC values being observed. Regarding kanamycin, the undoubted increase in the MIC value (32-fold) in the mutant strain is to be expected by the insertion of the aphA-3 cassette when constructing the mutant strain and proves the success of the insertional mutagenesis and natural transformation.

Concerning metals (Table 2), there was a noticeable increase in the susceptibility of the mutant strain to cadmium (eight-fold reduction in the MIC value), while for copper, manganese, zinc, and sodium, this increase is less explicitly, with the MIC values reduced to half of the values compared to the parent strain, suggesting that these compounds may also constitute possible substrates for this pump.

Overall, the results obtained for the growth assays of the parental and mutant strains of A. butzleri, in the presence of several potential substrates of the YbhFSR efflux pump (antibiotics, disinfectants, and heavy metals), indicated that this pump recognized a wide range of substrates and confirmed the role of this pump in the extrusion of the tested antimicrobials agents. The confirmation was demonstrated by the growth of the mutant strain in the presence of ampicillin, chloramphenicol, erythromycin, benzalkonium chloride, cadmium, copper, manganese, and zinc, which, compared to the growth of the parental strain, was inhibited or reduced (Figure S3).

**Figure 2:**
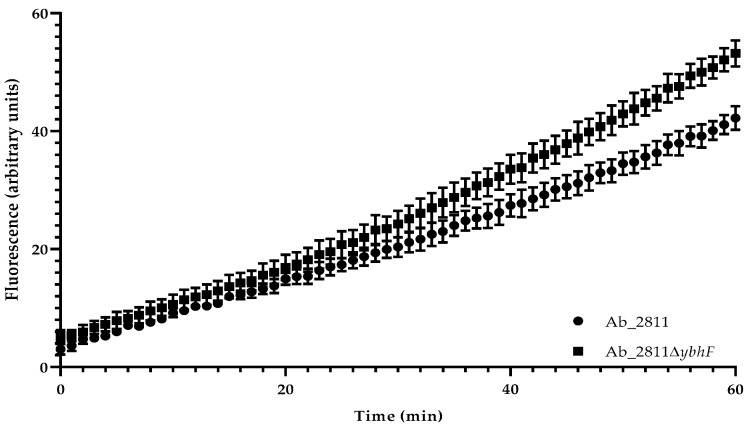
Accumulation of ethidium bromide along 60 min of incubation for parental strain *Aliarcobacter buzleri* Ab_2811 and mutant strain Ab_2811∆*ybhF*. Data match to mean ± standard error of the means (SEM) from three independent experiments.

To ascertain whether the increased susceptibility was associated with functional changes in the YbhFSR efflux system and was a consequence of the efflux loss, the intracellular accumulation of ethidium bromide assays was performed for parental and mutant strains (Figure 2). A time-dependent increase in fluorescence was observed for both strains, with a slight increase in the fluorescence over time for the YbhFSR mutant compared to the increased fluorescence shown by the native strain. This increment became more evident after minute 30, accentuating the tendency of the two curves to move apart and the difference in the fluorescence emitted by the two strains, suggesting the functional role of the YbhFSR pump in the *A. butzleri* efflux.

### 2.4. Effect of the Inactivation of YbhFSR Efflux Pump on Aliarcobacter butzleri Resistance to Oxidative Stress and Virulence

**Figure 3:**
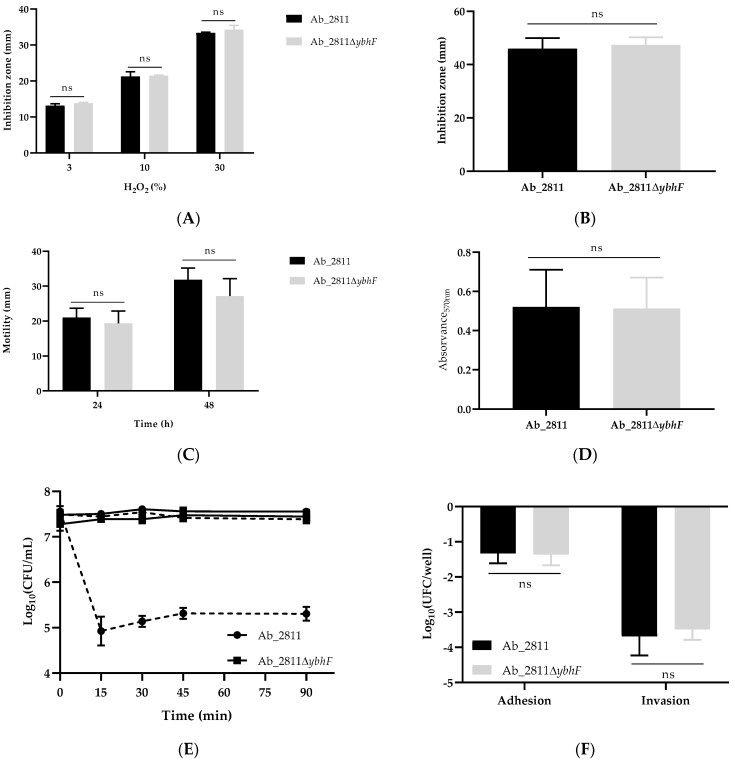
Evaluation of determinant virulence factors for parental strain *Aliarcobacter buzleri* Ab_2811 and mutant strain Ab_2811∆*ybhF*. Susceptibility to oxidative stress after 48 h of incubation, induced by (**A**) hydrogen peroxide and (**B**) methyl viologen. (**C**) Motility ability at 24 and 48 h of incubation. Data correspond to the mean ± SD. (**D**) Biofilm formation ability after 48 h of incubation, under microaerophilic conditions. (**E**) Susceptibility to human serum over 90 min of incubation, corresponding the solid lines to the incubation of bacteria with inactivated serum and dash lines with non-inactivated serum. (**F**) Adhesion and invasion assays in the Caco-2 cell line. Data correspond to the mean ± SEM considering at least three independent assays. ns: *p* > 0.05 by Student’s *t* test.

Regarding the evaluation of the potential functional role of the YbhFSR transporter in the resistance to oxidative stress (Figure 3A,B) and virulence (Figure 3C–F) of A. butzleri, no statistically significant differences were detected between the profiles of the mutant and parental strains for the several assays performed. The only exception was the susceptibility to human serum (Figure 3E), for which the parental strain *A. butzleri* Ab_2811 showed a significant reduction in the number of cultivable cells as early as 15 min of incubation, while the mutant strain Ab_2811∆*ybhF* was able to survive throughout the assay lasting 90 min, similarly to the control using inactivated serum, revealing a resistance profile to human serum (Figure 3E).

## 3. Discussion

The in silico analysis of the YbhFSR efflux pump showed that *ybhF* codes for a cytoplasmic export protein belonging to the ABC transporter family and represents the subunit containing the binding regions to ATP, which are responsible for the energy process of the YbhFSR efflux pump in *A. butzleri*. From the acquired results, the obtained structure ({TMD}_2_-{NBD}_2_) (Figure S1a) typically found in ABC transporters stands out, in which the NBD and TMD are encoded by different proteins [32]. Finally, the identification of the conserved domains of Walker A, Q-loop, and Walker B, and of the signature motifs of the D-loop and Switch motif (Figure S1b), represented important data in the analysis and supported the information described above. The results obtained are in agreement with the ones obtained by Feng et al. (2020) in *E. coli*, describing YbhF as an ATP-binding component with two NBDs, while YbhS and YbhR were identified as the transmembrane components [31].

Following the results obtained from the bioinformatic analysis, *ybhF* was the target gene for mutation. Coding for the subunit responsible for ATP-binding, whose energy released during its hydrolysis allows for the translocation of the substrates through the efflux pump, it is presumable that if this gene is deleted, the transporter loses its energy source and, consequently, the substrate transfer capacity. Thus, considering that the interruption of the gene responsible for its energy process and, consequently, essential for its function has been fulfilled (Figure S2), it becomes plausible to assume that the inactivation of the *ybhF* gene renders the YbhFSR efflux system non-functional. The *A. butzleri* Ab_2811 strain was selected for the construction of the mutant since it is a naturally transformable strain, harbors a copy of the operon YbhFSR, and displays a multidrug resistance profile [7,33,34].

Several studies have shown that the deletion of a gene belonging to an efflux pump can affect bacterial growth, pointing out as a possible justification the decrease in the extrusion of the toxic compounds out of the cell. This decrease in efflux will lead to its intracellular accumulation and, ultimately, to a difference in the bacterial growth profile between the parental and mutant strains [16]. Specifically on ABC-type transporters, the slower growth or more rapid entry into the death phase has been reported for mutant strains of the efflux systems belonging to this family in other bacterial species [35,36,37]. In contrast, other studies show that mutations in genes from the ABC family of efflux pumps do not affect the bacterial growth [36,38,39,40,41,42]. In this work, the mutant Ab_2811∆*ybhF* exhibited a growth curve similar to the parental strain (Figure 1), which is in line with these last results. Furthermore, the results obtained are consistent with those found in the mutants of efflux systems belonging to the RND superfamily in *A. butzleri*, where, despite a slight decrease in the growth of the constructed strains, identical growth profiles were found between the native and their mutant strains [15].

Therefore, an identical growth profile between the parental and the mutant strains was ensured and the contribution of the YbhFSR efflux pump to the *A. butzleri* resistance to different antimicrobial agents was assessed through the MIC values (Table 1 and Table 2), and also the growth curves in the presence of some of the included compounds were studied (Figure S3). The results obtained in this work are in line with several studies on ABC transporters in other bacterial species, where the inactivation of genes coding for the efflux pump subunits resulted in an increased susceptibility to different compounds. These results corroborate the previous reports on the role of MacAB from *Serratia marcescens* in the resistance to aminoglycoside antibiotics [40] or the contribution of the MacABCsm efflux system in the intrinsic resistance of aminoglycosides, macrolides, and polymyxins in *Stenotrophomonas maltophilia* [38].

The results obtained for the metals (Table 2) should be highlighted since the topic of resistance to metals in *A. butzleri* is largely unexplored. In addition to the ability to recognize a wide range of heavy metals, the results obtained for the sodium metal are in line with the study of the YbhFSR transporter in *E. coli*, which was shown to have a transport function of Na^+^ (Li^+^), namely being a Na^+^(Li^+^)/H^+^ antiporter [31]. Heavy metal resistance is the outcome of the overlay of diverse resistance systems that share substrates, where some of these widespread structures may be responsible for the extrusion of the excess of heavy metals [43]. Among the tested metals, iron and zinc are examples of important metals for the bacterial metabolism; nonetheless, in the presence of high metal concentrations, bacterial growth may be inhibited, involving the production of noxious reactive oxygen species or even the inadequate metalation of key metabolic pathway enzymes. The intracellular levels of metal ions are monitored by metal sensors that also balance the expression of several pathways, such as the efflux [44]. Regarding the metal resistance in *A. butzleri*, including heavy metals, two mechanisms are likely to function, one relying on the presence of the iron transport proteins FeoA and FeoB, and the other through the export of cadmium, zinc, and cobalt, involving the corresponding transporting of ATPase (*cadA*) and the metal/H+-K+ antiporter (*czcD*) [45,46].

Overall, despite the YbhFSR efflux pump seeming to recognize a broad range of substrates and demonstrating playing a role in the extrusion of compounds, it does not seem to play a significant role in the multidrug resistance of the *A. butzleri* Ab_2811 strain. Indeed, with the exception of benzalkonium chloride, ethidium bromide, and cadmium, for which a marked reduction in the MIC values was observed in the mutant strain, the reduction in the MIC values for most of the remaining compounds was slight, so ampicillin, erythromycin, chloramphenicol, acriflavine, copper, manganese, zinc, and sodium were only suggested as potential efflux pump substrates. Thus, a distinct contribution of different efflux pumps to the *A. butzleri* multidrug resistance is expected, with a more evident role of the RND efflux pumps, such as AreABC, as previously demonstrated [14,15].

Regarding the ethidium bromide accumulation assay, a decrease in the dye extrusion capacity and, consequently, an increase in the fluorescence presented by the mutant strain Ab2811∆*ybhF* compared to the native strain was verified (Figure 2). This observation confirmed the functional role of the YbhFSR pump in the *A. butzleri* efflux, although this is less relevant than the role demonstrated for efflux pumps belonging to the RND efflux pump superfamily [14,15].

In addition to their recognized role in the extrusion of various compounds, such as antibiotics, disinfectants, or even heavy metals, and in the acquisition of a resistance to these antimicrobial agents, efflux pumps are also involved in a varied number of bacterial physiological processes [16,40]. Among these, the extrusion of toxic metabolites and molecules associated with the quorum-sensing process, motility modulation, and biofilm formation are the most studied processes [15,47,48,49,50]. Additionally, the importance of studying other relevant virulence mechanisms can be highlighted, such as the susceptibility to human serum or the ability to adhere to and invade host cells, since *A. butzleri* needs these skills to colonize and establish a successful infection. In fact, Mateus et al. (2021) previously showed a significant contribution of three RND efflux systems to the virulence of *A. butzleri* [15].

Several studies have demonstrated an attenuation of virulence features or the ability to tolerate stress in the mutant bacterial strains of ABC-type efflux systems [38,40,51,52,53]. However, based on the profile drawn for the Ab_2811∆*ybhF* mutant strain (Figure 3), it can be concluded that the efflux system under study does not play a role in the virulence or adaptation, such as the susceptibility to oxidative stress, motility, biofilm formation capacity, or ability to adhere and invade Caco-2. Contrasting to these results, a role in the extrusion of oxidative stress-inducing agents, such as hydrogen peroxide, and in bacterial survival during the oxidative stress process, has been widely described for ABC transporters in several bacterial species, highlighting the involvement of efflux pumps in this protection process [38,40,53]. In addition, other authors have shown a significant reduction in the motility of the mutant strain of the MacAB efflux pump from *S. marcescens* [40] or, still focusing on the same system of efflux, the decrease in the biofilm formation capacity is associated with its inactivation in several bacterial species [38,40,52].

Adherence, invasion, and resistance to human serum are key mechanisms for bacterial pathogens to establish an infection. The present work went further in the investigation, for the first time, of the putative role of ABC transporters in these mechanisms. Although no significant differences in the ability to adhere and invade the host cells were noted between *A. butzleri* Ab_2811 and Ab_2811∆*ybhF* strains (Figure 3F), distinct survival profiles were observed for the assay with human serum (Figure 3E). Indeed, while the parental strain showed to be highly susceptible, the mutant strain was able to survive throughout the assay. Although further studies are needed to confirm this result and the underlying mechanism, it can be speculated that the inactivation of the YbhFSR efflux pump has a potential impact on the expression of porins with functions associated with binding to the molecules of the complement system, conferring the mutant strain with a resistance to human serum. The correlation between the expression of efflux pumps and porins in several bacterial species has already been demonstrated [54,55]. In addition, the virulence mechanism associated with the modification of the porin expression, strategically involved in inducing damage to host cells, was recently described in *Acinetobacter baumannii* [56]. In *A. baumannii*, the importance of porin OmpA in multiple functions has been described, among which stands out its binding to the H factor present in human serum, avoiding bacterial kill by the complement system [56,57]. Besides this mechanism, other porins may also contribute by inhibiting the alternative complement system’s activation and influence the development of a resistance to serum [58,59].

In sum, the role of the YbhFSR efflux pump in the antimicrobial resistance of *A. butzleri* was evidenced, which, on the other hand, did not demonstrate to have a relevant functional role in the virulence of this microorganism. This study contributed with important data to a better understanding of the resistance and virulence mechanisms of the enteric pathogen *A. butzleri.*

## 4. Materials and Methods

### 4.1. Bacterial Strains and Growth Conditions

The parental strain for this study corresponded to the *A. butzleri* strain Ab_2811, isolated from a poultry carcass neck skin by [34]. Bacterial cells were preserved in brain heart infusion (BHI, Liofilchem, Via Scozia, Italy) containing 20% glycerol at –80 °C. When needed, the strains were inoculated in tryptone soy agar (TSA, VWR, Leuven, Belgium) and incubated under aerobic conditions at 30 °C for 24 to 48 h. After incubation, the inoculated plates were stored at 4 °C for a maximum period of two weeks. Before each assay, the strains were previously inoculated in TSA medium for 24 h at 30 °C under aerobic conditions. For growth in broth culture, the strains were transferred to 10 mL of tryptic soy broth (TSB, VWR, Leuven, Belgium) with an initial optical density at 620 nm (OD_620 nm_) of 0.02 and incubated for 16 h on an orbital shaker at 30 °C and 100 rpm, under aerobic conditions.

### 4.2. Bioinformatic Analysis of YbhFSR Efflux Pump

Before starting the bioinformatic analysis, amino acid sequences were retrieved with the ExPASY tool (https://web.expasy.org/translate/) using the gene sequences as the input (ENA accession number ERR3523203, with locus tag ABU_RS06805, ABU_RS06810 and ABU_RS06815) [7]. The location and size of the proteins constituting the efflux pump under study were acquired using SOSUIGramN software (https://harrier.nagahama-i-bio.ac.jp/sosui/sosuigramn/sosuigramn_submit.html), and the DeepTMHMM program (https://dtu.biolib.com/DeepTMHMM) allowed for a prediction of its structure. The basic physicochemical properties of the YbhF protein were predicted using Protparam software (https://web.expasy.org/protparam/). To predict the type of protein, the YbhF sequence was tested in the online software Batch Web CD-Search tool (NCBI) (https://www.ncbi.nlm.nih.gov/Structure/bwrpsb/bwrpsb.cgi), and the identification of its conserved domains was performed using the online Conserved Domains tool (NCBI) (https://www.ncbi.nlm.nih.gov/Structure/cdd/wrpsb.cgi).

### 4.3. Construction of Aliarcobacter butzleri Ab_2811ΔybhF Mutant Strain

The construction of the *A. butzleri* efflux pump mutant was performed using insertional mutagenesis, where the *ybhF* gene, responsible for ATP-binding, was interrupted by a kanamycin resistance cassette (*aphA-3*). The *aphA-3* cassette was obtained by the enzymatic digestion of pUC18-K2 with BamHI and KpnI, followed by purification. Thus, it was necessary to construct an exogenous DNA fragment containing regions homologous to the regions downstream of the start codon and upstream of the stop codon of the *ybhF* gene, flanking the *aphA-3* cassette, and its incorporation into the bacterial genome by the process of natural transformation and homologous recombination, as previously described, with minor modifications [33]. For this, specific primers were designed and are described in Table S1. Briefly, *A. butzleri* Ab_2811 was cultured in TSA supplemented with 5% (*v/v*) of defibrinated horse blood at 30 °C for 24 h in microaerophilic conditions. After incubation, a suspension was prepared with 5 × 10^9^ CFU/mL in 200 µL of TSB, used to inoculate a new plate of the same medium and under the same conditions for 4 h. Then, about 2.5 µg of the transformant DNA was added and the plate was incubated for 8 h. At this point, the culture was transferred to another TSA plate and incubated for 18 h. After this incubation period, the biomass was transferred to TSB and applied to a TSA plate supplemented with defibrinated horse blood and 50 µg/mL of kanamycin and incubated for 5 days under the above conditions, time after which it was observed for the presence of the representative colonies of mutants. The genomic DNA from a few mutant colonies selected in the last step of the natural transformation process was used for the amplification of the gene region using the primers designated in Table S1. As a control, genomic DNA from the parental strain under study was used. The transformation was confirmed by the variation in the size of the amplification of the gene region in the parental and mutant strain by the PCR technique, and, complementary, by the Sanger method bidirectional sequencing of the *ybhF* gene region in the mutant strain using ybhF_A1 and ybhF_B2 primers (Table S1).

### 4.4. Determination of Growth Curves of Parental and Mutant Strains

The strains grew as previously mentioned and then they were cultured in TSB starting with an OD_620 nm_ of 0.02 at 30 °C, 100 rpm. Throughout the assay, samples were collected from each culture every 2 h and OD_620 nm_ readings were taken until the stationary phase of the two strains was reached, with the last sample being read at 24 h. This procedure was performed at least two times independently.

### 4.5. Antimicrobial Susceptibility Test

The MIC was determined by an agar dilution method for antibiotics, including ampicillin, cefotaxime, kanamycin, ciprofloxacin, erythromycin, and chloramphenicol; benzalkonium chloride and chlorhexidine as disinfectants; acriflavine as a germicide; ethidium bromide as a substrate for efflux pumps; and metals, especially heavy metals, as previously described by Isidro et al. (2020), with minor modifications [7]. Briefly, double dilutions of decreasing concentrations of each antimicrobial agent were incorporated in TSA plates which were inoculated with 2 µL of each inoculum, after a turbidity adjustment to 0.5 McFarland and a dilution (1:10) in a saline solution of 0.85% NaCl (*w/v*. After inoculation, plates were incubated for 48 h at 30 °C under aerobic conditions. The MIC was defined as the lower concentration of each compound that ceased the growth of the strain [60]. This assay was performed in at least three independent assays.

### 4.6. Growth Tests for Compounds Tolerance of Aliarcobacter butzleri

The compounds that demonstrated a decrease in the MIC in the mutant strain compared to the MIC obtained for the parent strain were selected for growth testing. For this, the growth of the two strains was monitored for 24 h with OD_620 nm_ readings taken every hour under the conditions previously described in 96-well microplates in a final volume of 200 µL with different concentrations of each compound in TSB.

### 4.7. Ethidium Bromide Accumulation Assay

The evaluation of the EtBr accumulation profile for the parental and mutant strains was based on a previously described protocol, with slight modifications [14]. The cells were harvested at the mid-exponential growth phase cultures of each strain by centrifugation, washed with phosphate-buffered saline (PBS, Lonza, Walkersville, MD, USA), and resuspended at OD_620 nm_ of 0.4. The suspension was transferred in triplicate to a black 96-well plate with a clear bottom (Greiner Bio-One, Frickenhausen, German), which was incubated for 10 min at 30 °C. EtBr was added to each well to a final concentration of 2 µg/mL and immediately after its addition, the fluorescence measurement of the samples was started using a Spectramax Gemini XS spectrofluorometer (Molecular Devices LLC, San Jose, CA, USA), with readings taken every minute, for 1 h, at excitation and emission wavelengths of 530 and 600 nm, respectively. Wells with the cellular suspension and PBS were included as a cell autofluorescence control, while wells with PBS and ethidium bromide solution to a final concentration of 2 µg/mL were used as ethidium bromide fluorescence control. Each assay was performed in triplicate and in three independent assays.

### 4.8. Stress Susceptibility Assay

The oxidative stress susceptibility, induced by hydrogen peroxide and methyl viologen, was determined by the disk diffusion assays as formerly described [15]. TSA plates were inoculated with a cellular suspension adjusted to OD_620 nm_ of 0.2 using a cotton swab, and 6 mm sterile paper disks containing 5 µL of 3%, 10%, and 30% hydrogen peroxide (LABKEM, Barcelona, Spain) and 5 µL of 125 mM methyl viologen (Sigma-Aldrich, St. Louis, MO, USA) were spotted. The plates were incubated at 30 °C in an aerobic atmosphere for 48 h, the time after which the bacterial growth inhibition halos were measured. These assays were performed three independent times. 

### 4.9. Motility Assay

The motility of the parental and mutant strains was performed according to the protocol previously described by Ferreira et al. (2018) [13]. The *A. butzleri* cells were collected and resuspended in TSB to an OD_620 nm_ of 0.02. Following, 5 µL of each strain was inoculated by stabbing the center of a semisolid TSA (0.4% agar) plate. The plates were incubated at 30 °C for 48 h under aerobic conditions, and the motility halos of the strains were measured at 24 and 48 h. This assay was performed at least three independent times.

### 4.10. Biofilm Formation Ability

To determine the ability of *A. butzleri* strains to form the biofilm, a previously described protocol was followed with few modifications [15]. The suspensions of the two strains under study were prepared by diluting an overnight culture to an OD_620 nm_ of 0.2, which was used to inoculate 24-well polystyrene plates (VWR, Belgium) with 1 mL of each suspension. Wells with only a medium were used as the negative control. After 48 h of incubation at 30 °C in microaerophilic conditions, the medium was removed, and the wells were dried for 1 h at 55 °C. After that, the biofilm was stained with 1 mL of crystal violet (AMRESCO, Leuven, Belgium) at 0.1% (*w/v*) in deionized water and incubated for 15 min at room temperature, followed by removing the unbound crystal violet by the washing of the wells three times with distilled water. The wells were dried at 55 °C for 15 min and then the bound crystal violet was solubilized with a 30% methanol and 10% acetic acid solution. Finally, to quantify the biofilm formation, the absorbance at 570 nm was determined using a microplate reader (Biorad, xMark) [15]. The assay was performed with eight replicates in at least three independent assays.

### 4.11. Serum Susceptibility Assay

The serum bactericidal assay was performed as previously described, with some adjustments [61]. Blood was collected from two healthy volunteer donors, left to clot at room temperature for 30 min, and then the serum was separated by centrifugation at 2000 rpm for 10 min at 4 °C, pooled and frozen in aliquots at −80 °C in sterile cryogenic tubes until use. The cells suspensions of the parental and mutant strains were prepared at a final concentration of approximately 10^7^ CFU/mL in TSB with 90% of serum. As a control, serum was replaced with inactivated serum. The assay was performed at 30 °C, viable counts of *A. butzleri* were determined at 0, 15, 30, 45, and 90 min of exposure to the serum by serial dilution in TSB followed by drop-count plating in TSA, and incubation took place for 48 h at 30 °C in aerobic conditions. This assay was realized three independent times.

### 4.12. Adhesion and Invasion Assay in a Caco-2 Cell Line

In vitro adhesion and invasion assays were evaluated according to the protocol described by Ferreira et al. 2014, with slight adaptations [62]. The Caco-2 human intestinal epithelial cells were grown in tissue culture flasks in Dulbecco’s modified Eagle medium high glucose (Sigma-Aldrich, USA) at a pH value of 7.2 supplemented with 10% (*v/v*) fetal bovine serum (PAN-Biotech, Aidenbach, Germany), 1% (*v/v*) nonessential amino acids (Lonza, USA), 100 µg/mL of streptomycin, and 100 U/mL of penicillin (Sigma-Aldrich, USA). The tissue culture flasks were incubated at 37 °C in a 5% CO_2_ humidified atmosphere, with the replacement of the medium every two days until the cells reached a semi confluent state of about 80%. Before the experiments, 24-well plates were seeded with 9 × 10^4^ cells/well and were incubated for 7 days at 37 °C and under the atmospheric conditions previously mentioned. After bacterial strains were grown as described above, collected, and washed, the *A. butzleri* cells were resuspended in the medium used for Caco-2 cell culture, without antibiotics. To allow for the occurrence of adhesion and invasion, monolayers were infected at a multiplicity of infection of approximately 100 and incubated for 3 h in the same atmospheric conditions. Following this period, the cells were washed three times to remove unbound bacteria and the number of interacting (adherent and internalized) bacteria was determined by lysing the Caco-2 cells with Triton X-100 (Sigma-Aldrich, USA) at 0.1% (*v/v*), followed by a plate count of the bacteria. For the evaluation of the bacterial invasion of Caco-2 cells, after 3 h of incubation, the extracellular bacteria were killed by incubating with 125 µg/mL of gentamicin (Sigma-Aldrich, USA) for 1 h. Then, Caco-2 cells were washed three times and lysed with 0.1% (*v/v*) Triton X-100, followed by plating serial dilutions of the lysates in TSA plates to determine the released intracellular bacteria. The plates used for bacterial counts in the adhesion and invasion assay were incubated under aerobic conditions at 30 °C for 48 h. Each assay was performed in triplicate at least three independent times.

## Figures and Tables

**Table 1:** Minimum inhibitory concentration, in µg/mL, of several antibiotic classes, disinfectants, germicide, and substrates of efflux pumps for the parental and derived mutant *Aliarcobacter butzleri* strains.

	MIC (µg/mL) (Fold Increase/Decrease)	
	Ab_2811	Ab_2811∆*ybhF*
Ampicillin	64	**32 (2)**
Cefotaxime	32	32 (ND)
Kanamycin	2	**64 (32)**
Gentamycin	0.5	0.5 (ND)
Ciprofloxacin	16	16 (ND)
Erythromycin	32	**16 (2)**
Chloramphenicol	128	**64 (2)**
Benzalkonium chloride	64	**16 (4)**
Chlorhexidine	1	1 (ND)
Acriflavine	32	**16 (2)**
Ethidium bromide	64	**16 (4)**

ND: No observed MIC difference. Changes of at least 2-fold are indicated in bold type.

**Table 2:** Minimum inhibitory concentration, in mM, of metals for the parental and respective mutant strains of *Aliarcobacter butzleri*.

	MIC (mM) (Fold Decrease)	
	Ab_2811	Ab_2811∆*ybhF*
Cadmium	0.25	**0.03 (8)**
Lead	8	8 (ND)
Cobalt	1	1 (ND)
Copper	0.5	**0.25 (2)**
Chrome	0.01	0.01 (ND)
Manganese	4	**2 (2)**
Mercury	0.003	0.003 (ND)
Molybdenum	64	64 (ND)
Nickel	1	1 (ND)
Silver	0.01	0.01 (ND)
Zinc	1	**0.5 (2)**
Lithium	32	32 (ND)
Sodium	256	**128 (2)**

ND: No observed MIC difference. Changes of at least 2-fold are indicated in bold type.

## Data Availability

Data are contained within the text and the Supplementary Materials.

## Supplementary Materials

The following supporting information can be downloaded at: https://www.mdpi.com/article/10.3390/antibiotics12020339/s1, Figure S1. Bioinformatic Analysis of the YbhFSR Efflux System. (a) Prediction of YbhF (1), YbhS (2) and YbhR (3) transmembrane domains using DeepTMHMM software. (b) Representation of the YbhF conserved domains analysis, through the software Conserved Domains, NCBI; Figure S2. Confirmation of the mutant constructed by PCR technique. Lane A: PCR product using specific primers for *ybhF* for the parental strain Ab_2811; Lane B: PCR product using specific primers for *ybhF* for the mutant Ab_2811Δ*ybhF* strain; M: Molecular weight; Figure S3. Growth tests for parental strain *Aliarcobacter buzleri* Ab_2811 and respective mutant strain Ab_2811∆*ybhF*. (a) 16 µg/mL Ampicillin; (b) 16 µg/mL Chloramphenicol; (c) 1 µg/mL Erythromycin; (d) 8 µg/mL Benzalkonium chloride; (e) 0.125 mM CdCl_2_; (f) 0.25 mM CuCl_2_·2H_2_O; (g) 0.25 mM Cl_2_Mn·4H_2_O; (h) 0.5 mM O_4_SZn·7H_2_O; Table S1. Identification of the primers used for amplification of the upstream and downstream regions of *ybhF*.
