# Supplementary material for: Putative Role of an ABC Efflux System in Aliarcobacter butzleri Resistance and Virulence
Source: Antibiotics (Basel). 2023 Feb 6;12(2):339. doi: 10.3390/antibiotics12020339 (PMC9951867; doi:10.3390/antibiotics12020339)
Supplement: Supplementary file 1 [file antibiotics-12-00339-s001.zip › antibiotics-2145146-supplementary.pdf]

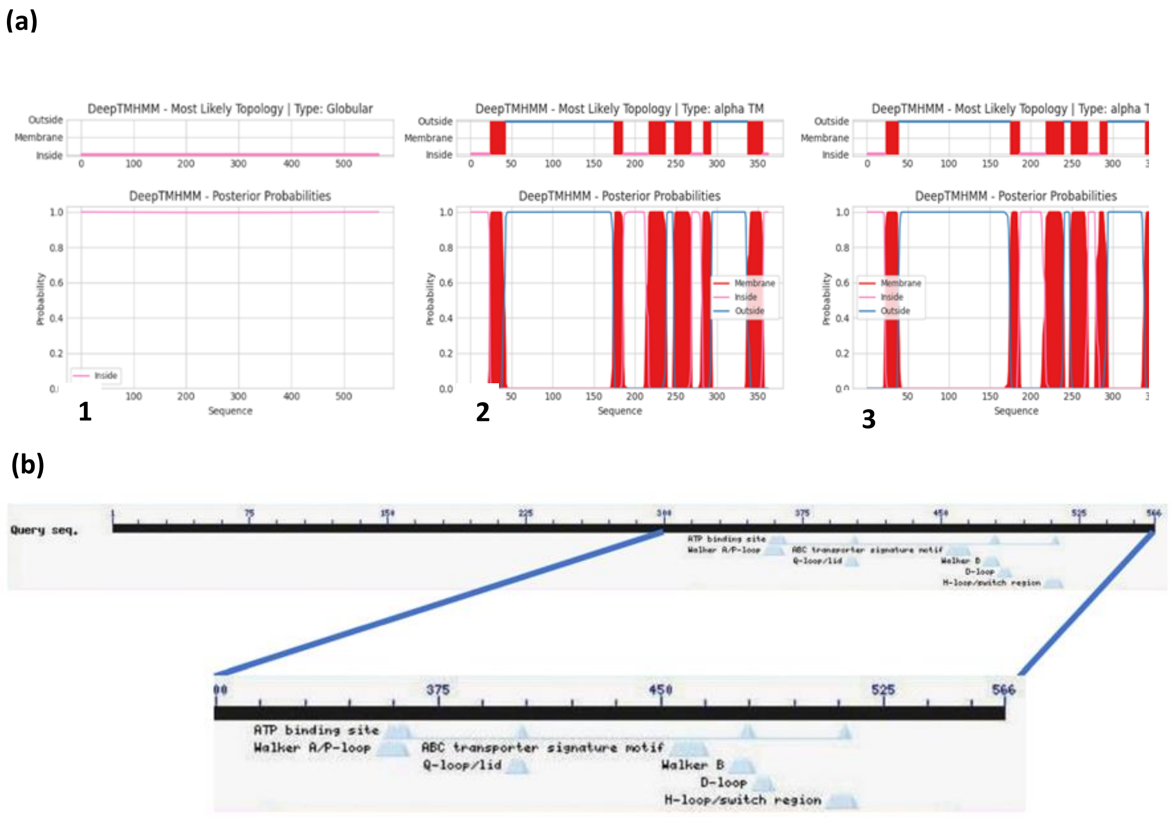

Figure S1. Bioinformatics Analysis of the YbhFSR Efflux System. (**a**) Prediction of YbhF (1), YbhS (2) and YbhR (3) transmembrane domains using DeepTMHMM software. (**b**) Representation of the YbhF conserved domains analysis, through the software Conserved Domains, NCBI.

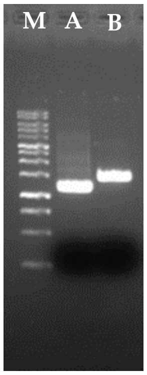

Figure S2. Confirmation of the mutant constructed by PCR technique. Lane A: PCR product using specific primers for *ybhF* for the parental strain Ab_2811; Lane B: PCR product using specific primers for *ybhF* for the mutant Ab_2811Δ*ybhF* strain; M: Molecular weight.

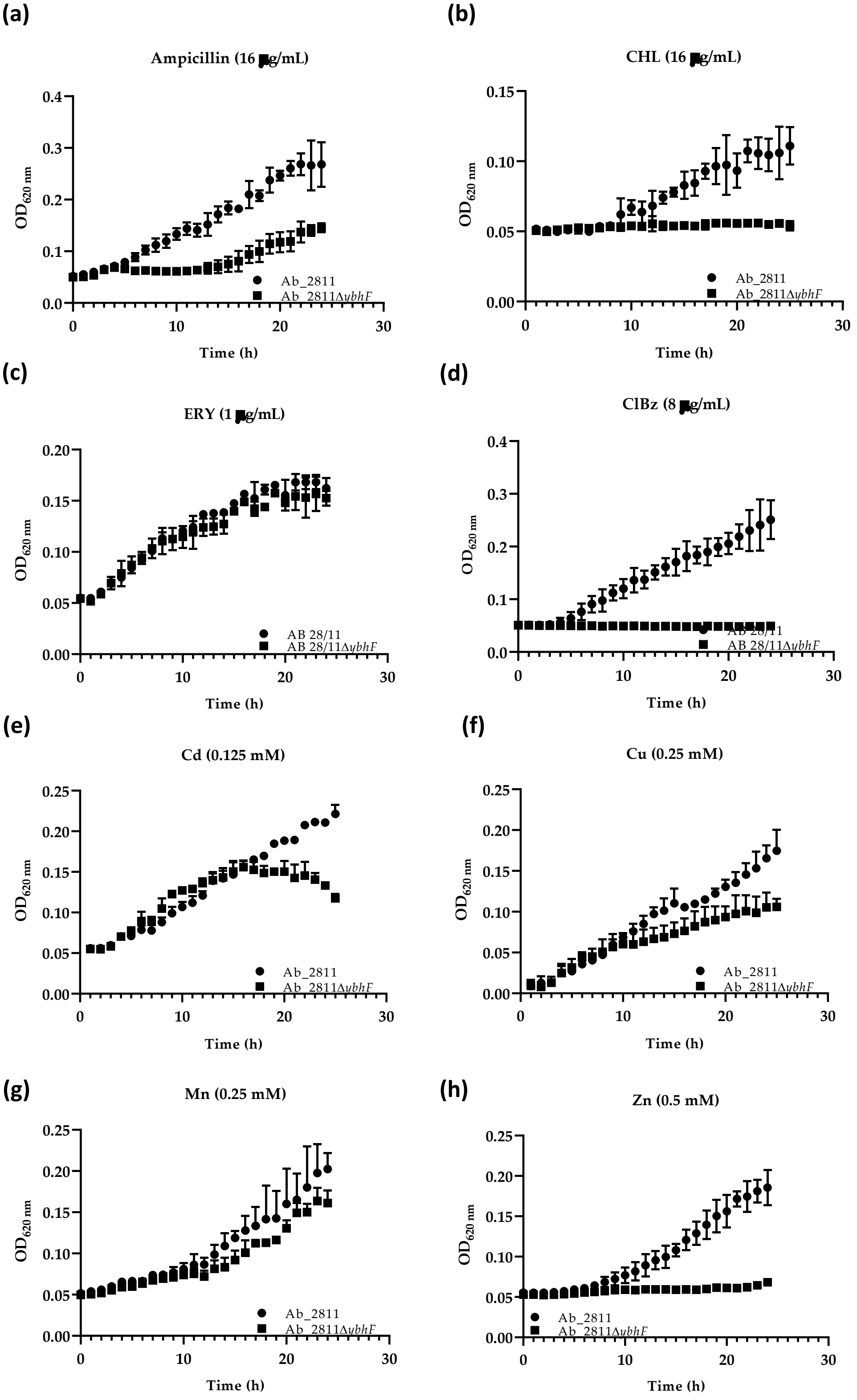

Figure S3. Growth tests for parental strain *Aliarcobacter buzleri* Ab_2811 and respective mutant strain Ab_2811Δ*ybhF*. (**a**) 16 µg/mL Ampicillin; (**b**) 16 µg/mL Chloramphenicol; (**c**) 1 µg/mL Erythromycin; (**d**) 8 µg/mL Benzalkonium chloride; (**e**) 0.125 mM $CdCl_2$; (**f**) 0.25 mM $CuCl_2.2H_2O$; (**g**) 0.25 mM $Cl_2Mn.4H_2O$; (**h**) 0.5 mM $O_4SZn.7H_2O$.

Table S1. Identification of the primers used for amplification of the upstream and downstream regions of *ybhF*.

Identification of primers	Sequence
ybhF_A1	5′-AGC CCC TAC TTT TGG TGA ATT-3′
ybhF_A2	5′-*TTA TTC CTC CTA GTT AGT CA*T CAC AAT TTT GAG CCT CAT CCA-3′
ybhF_B1	5′-*TAC CTG GAG GGA ATA ATG A*AC TCT ATT GGT TAT ATG GCG CA-3′
ybhF_B2	5′-ACA GAA CAT GCA AGT GAG AGT C-3′

Note: Adapter sequences for binding to the *aphA-3* resistance cassette were used and identified in italics.
